# Supplementary material for: Pyrazinamide and derivatives block ethylene biosynthesis by inhibiting ACC oxidase
Source: Nat Commun. 2017 Jun 12;8:15758. doi: 10.1038/ncomms15758 (PMC5472784; doi:10.1038/ncomms15758)
Supplement: Supplementary Information — Supplementary Figures [file ncomms15758-s1.pdf]

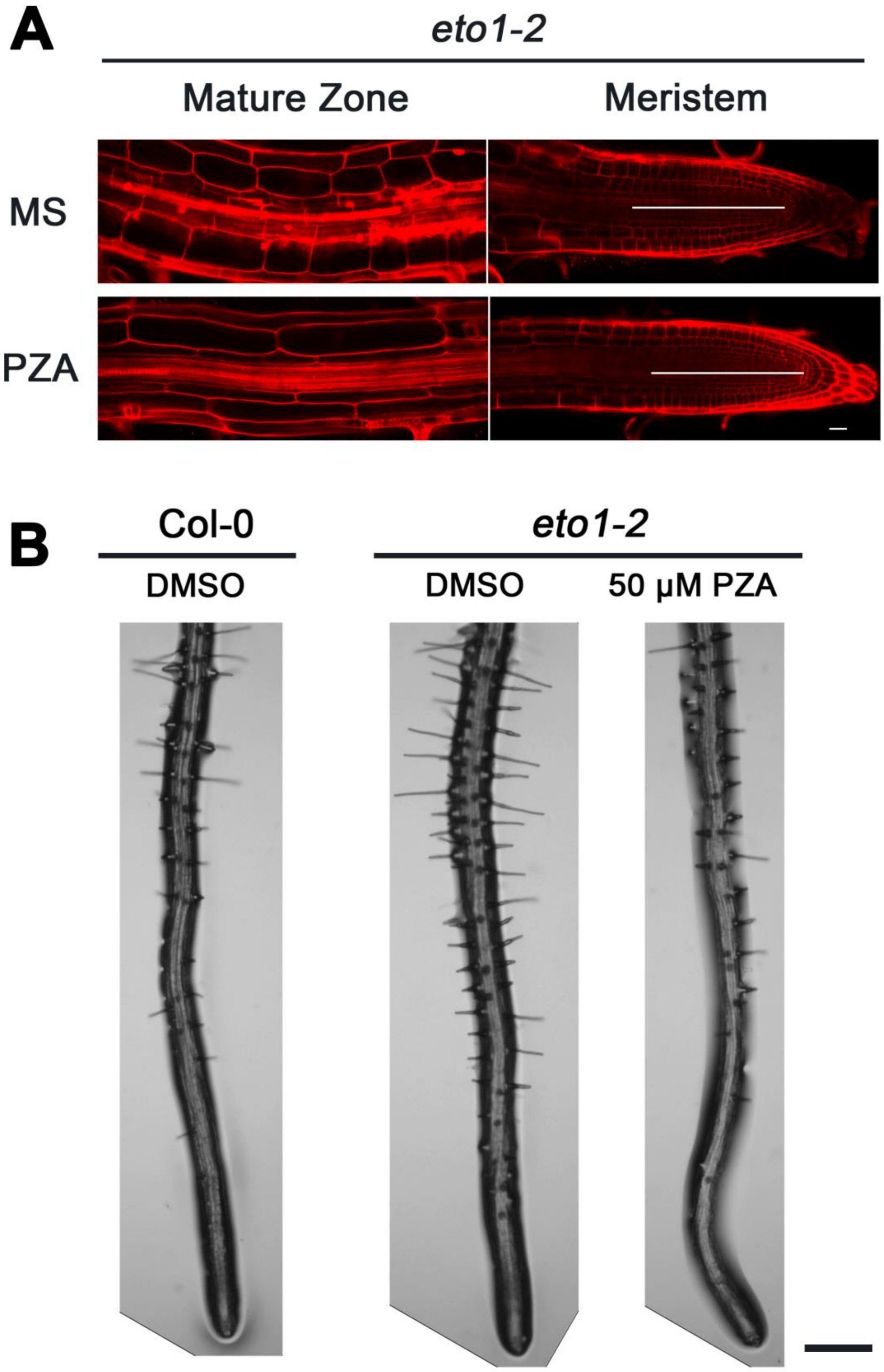

Supplementary Figure 1 PZA inhibits root hair formation as well as cell elongation in the maturation zone of *eto1-2* roots.

(**A**) The PI staining of the roots of three-day-old etiolated seedlings of Col-0 and *eto1-2* grown on MS medium supplemented with or without PZA (50 μM). White bars indicate the length of the meristem. Bar = 2 μm.

(**B**) Three-day-old etiolated seedlings grown on MS medium supplemented with or without PZA (50 μM), indicative of root hair formation. Bar = 200 μm.

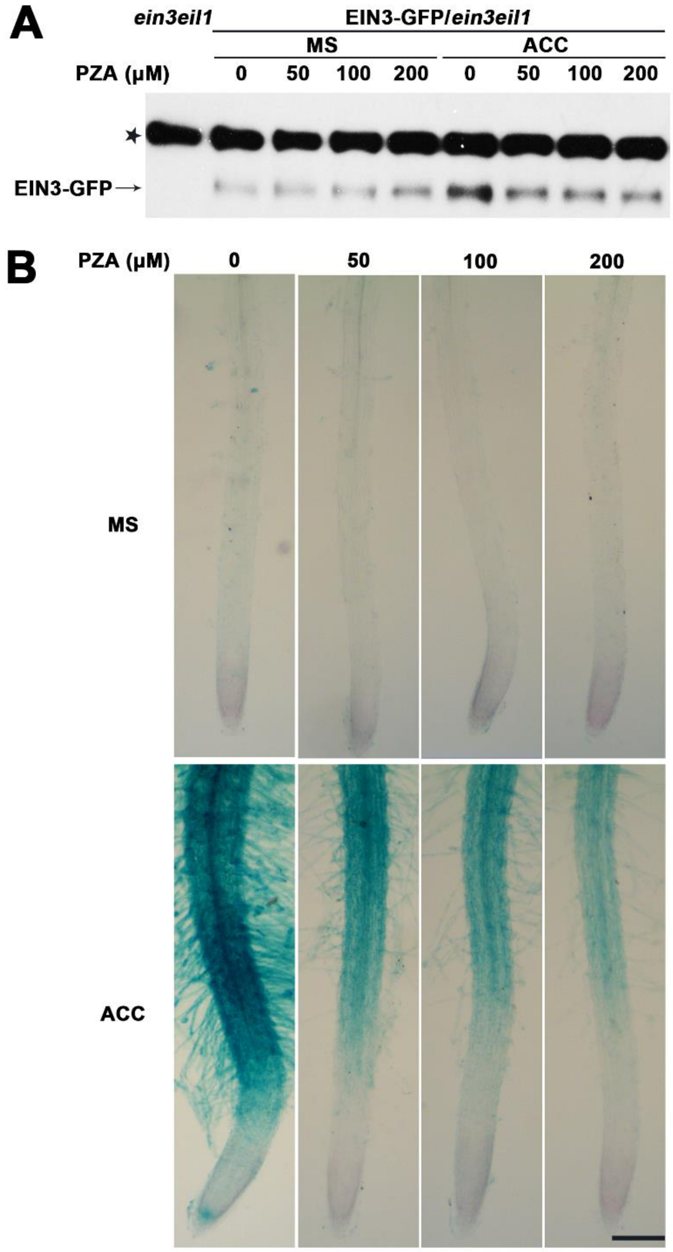

Supplementary Figure 2 PZA inhibits the ACC-induced EIN3 protein accumulation and transcriptional activation of EIN3 target promoter.

(**A**) EIN3 protein accumulation under different treatments. Three-day-old etiolated seedlings of EIN3-GFP/*ein3eil1* grown on MS medium supplemented with 1 μM ACC and/or different concentrations of PZA (50, 100 and 200 μM). The protein level of EIN3-GFP of each treatment was analyzed by Western blot using α-GFP antibody. Arrow indicates the band of EIN3-GFP. Star indicates a non-specific band serving a loading control.

(**B**) The GUS staining of *pEBS:GUS* in three-day-old etiolate seedlings of Col-0 treated with 1 μM ACC and/or different concentrations of PZA (50, 100 and 200 μM). Bar = 200 μm.

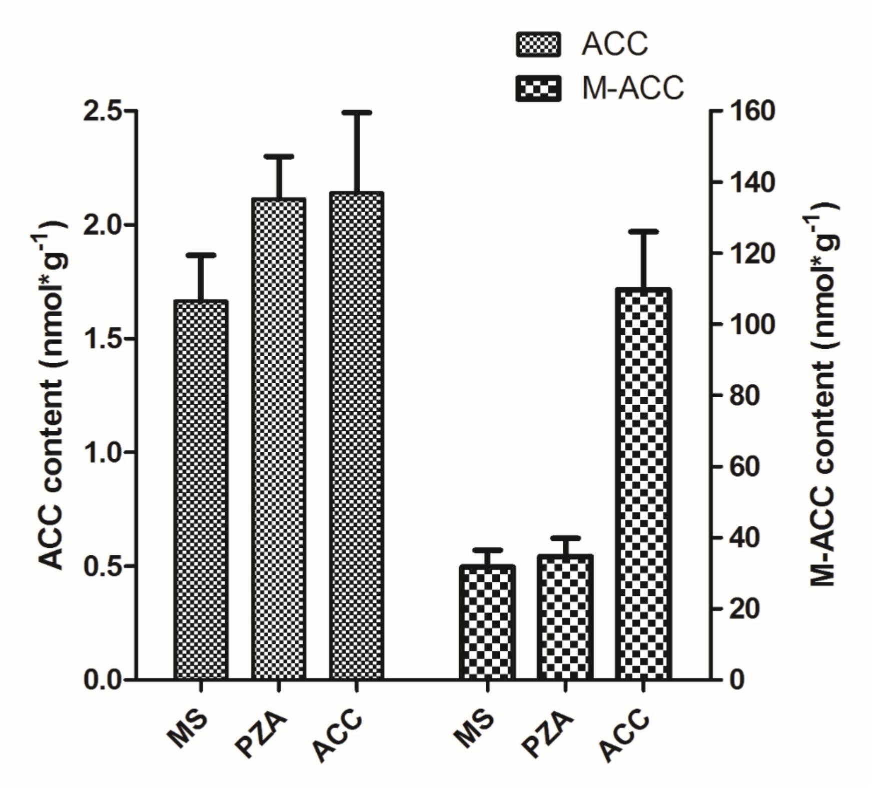

Supplementary Figure 3 PZA has no effect on the ACC conjugating enzymes.

Ten-day-old green seedlings of *eto1-2* grown on MS medium supplemented with or without 50 µM PZA or 1 µM ACC. The ACC or M-ACC level of each treatment was measured and analyzed according to the method. Bars represent the means (± S.D.) of three independent treatments

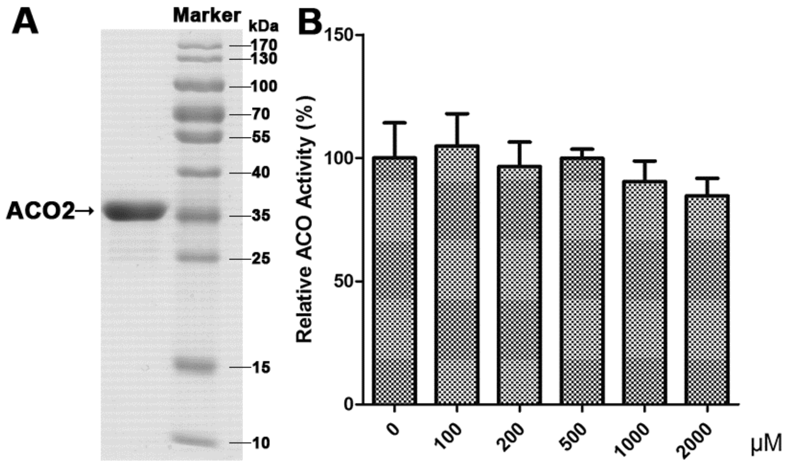

Supplementary Figure 4 PZA does not inhibit ACO activity *in vitro*.

(**A**) A Coomassie-stained gel depicting the purified AtACO2 protein.
(**B**) Quantification of the relative AtACO2 activity (compared to ACC application) treated with different concentrations of PZA as indicated. Bars represent the means (± S.D.) of three independent replicates, and the experiment was carried out twice with similar results.

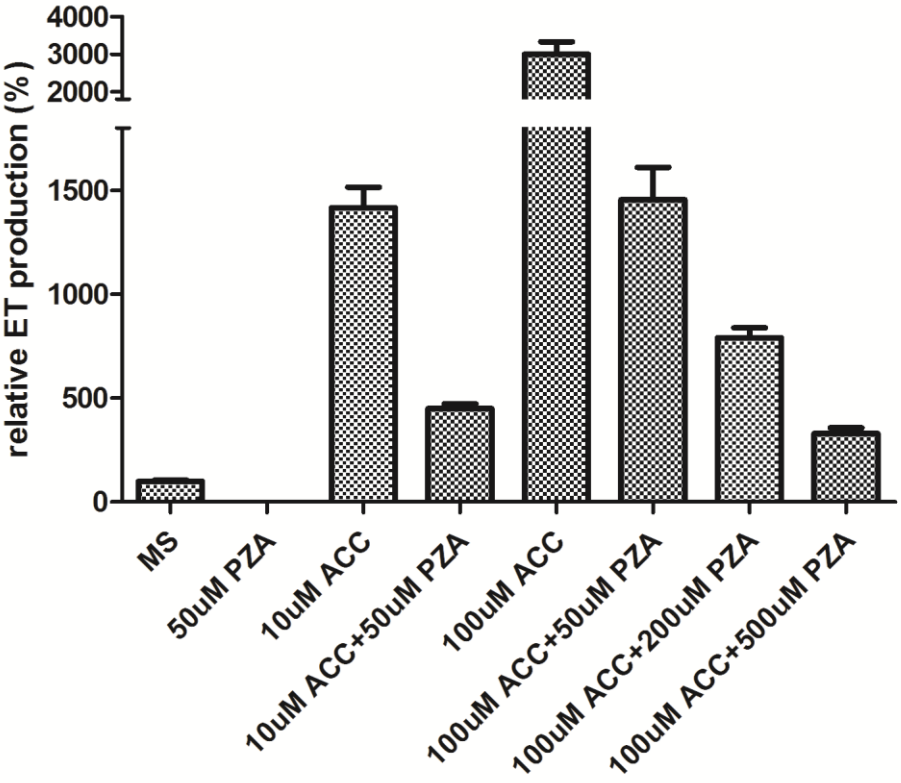

Supplementary Figure 5 PZA inhibits ethylene production in *Arabidopsis* suspension cultured cells.

Quantification of the relative ethylene production (compared to MS treatment) of suspension cultured cells of *Arabidopsis* with different treatments indicated in the figure. Bars represent the average relative ethylene (ET) production (± S.D.) of three independent treatments.

sample

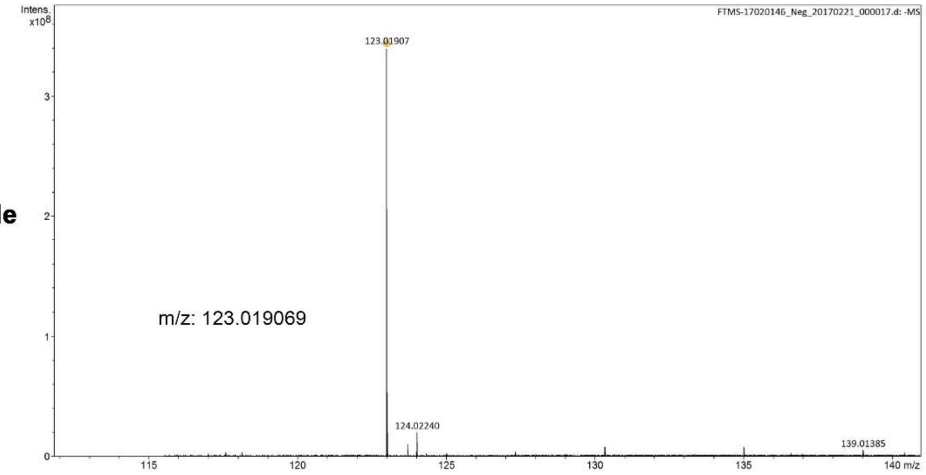

Meas. m/z	#	Ion Formula	Score	m/z	err [ppm]	Mean err [ppm]	mSigma	rdb	e⁻ Conf	N-Rule
123.019069	1	C5H3N2O2	100.00	123.018904	-1.3	7.7	3.0	5.0	even	ok

negative control

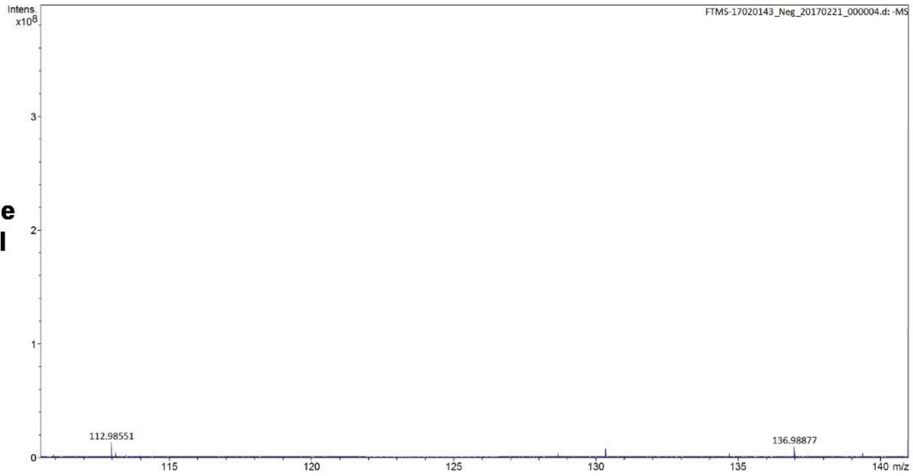

POA reference

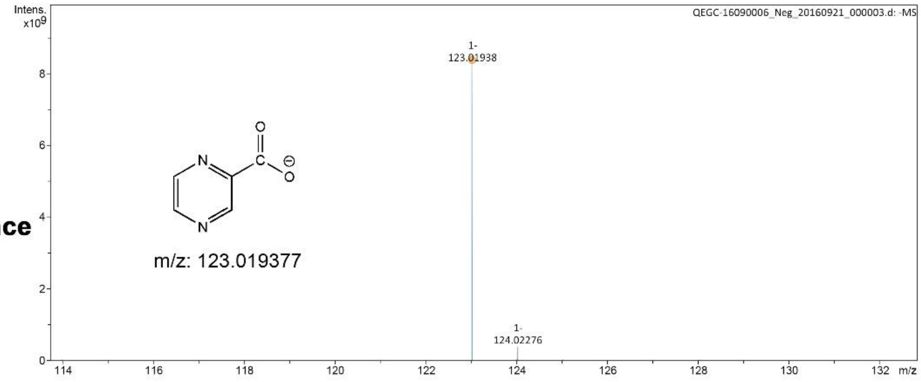

Meas. m/z	#	Ion Formula	Score	m/z	err [ppm]	Mean err [ppm]	mSigma	rdb	e⁻ Conf	N-Rule
123.019377	1	C5H3N2O2	100.00	123.018904	-3.8	4.9	4.0	5.0	even	ok

PZA reference

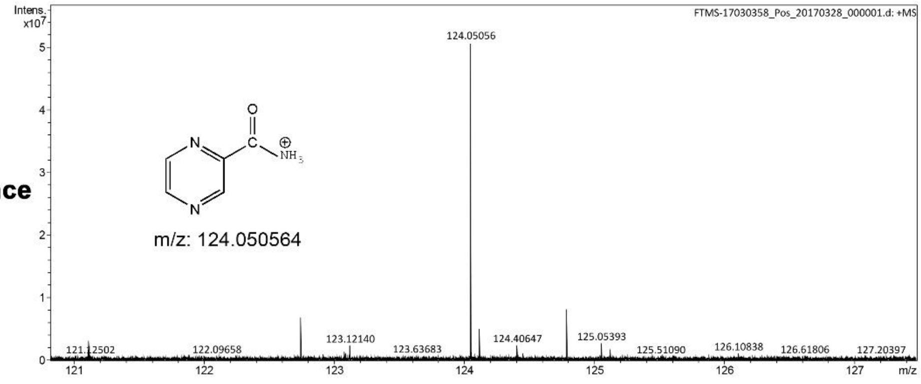

Meas. m/z	#	Ion Formula	Score	m/z	err [ppm]	Mean err [ppm]	mSigma	rdb	e⁻ Conf	N-Rule
124.050564	1	C5H6N3O	100.00	124.050538	-0.2	-0.2	6.3	5.0	even	ok

Supplementary Figure 6 PZA can be converted into POA in *Arabidopsis* suspension cells

Mass Spectrometry analysis of POA in *Arabidopsis* suspension cells. Cells were cultured in liquid MS (**negative control**) or MS medium supplemented with 500 μM PZA (**sample**) for 24 hours. The culture supernatant was collected by centrifugation and treated with NaOH (pH > 11) followed by extracting with ethyl acetate twice to eliminate lipid-soluble molecules. The aqueous phase was collected and treated with HCl (pH < 2), and protonated cultures were extracted with ethyl acetate five times to enrich POA. The resulting samples were concentrated by reduced pressure distillation, and subjected to MS analysis. The peak for POA (m/z = 123.0189) is showed as indicated. For data presentation clarity, we only include spectrums of m/z ranging from 110-140 that covers the peak of POA. The Mass Spectrometry profile of the POA standard (POA reference) and PZA standard (PZA reference) are shown for comparison.

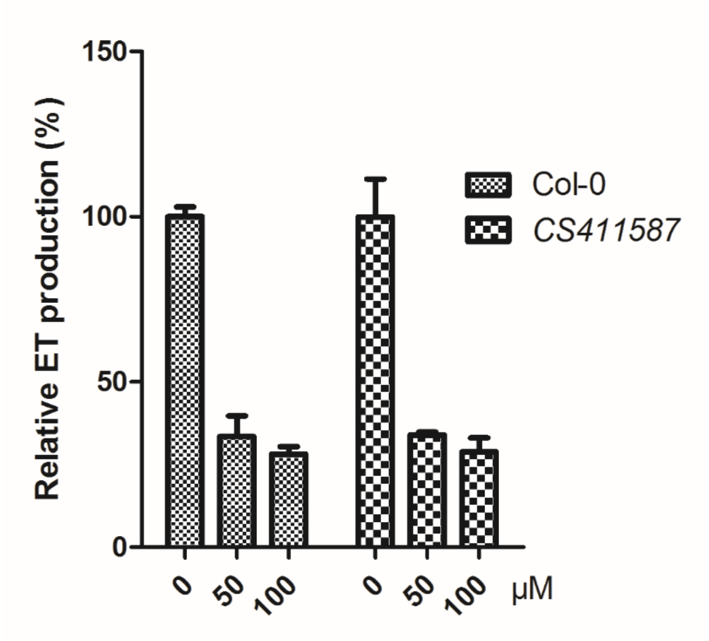

Supplementary Figure 7 Mutation of NIC2 shows no obvious effect on PZA inhibition.

Quantification of the relative ethylene production (compared to Col-0 under MS treatment) of Col-0 and CS411587 (NIC2 single T-DNA insertion mutant) with different concentration of PZA. Bars represent the average relative ethylene (ET) production (± S.D.) of three independent treatments.

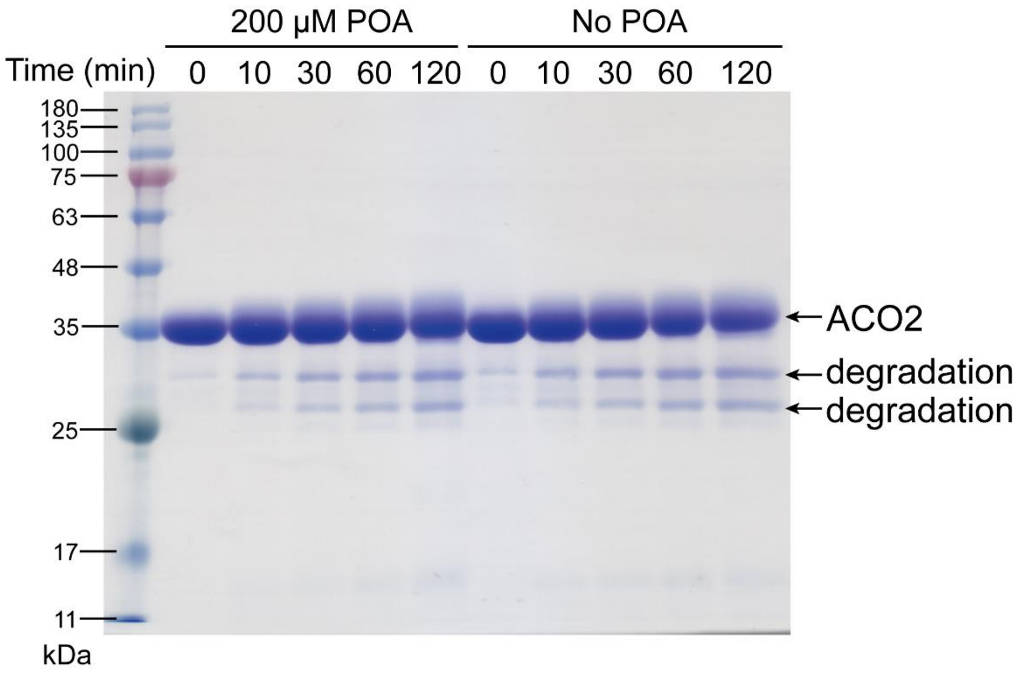

Supplementary Figure 8 POA does not influence the stability of purified AtACO2.

AtACO2 was incubated with or without POA for different durations. POA did not obviously change the stability AtACO2.

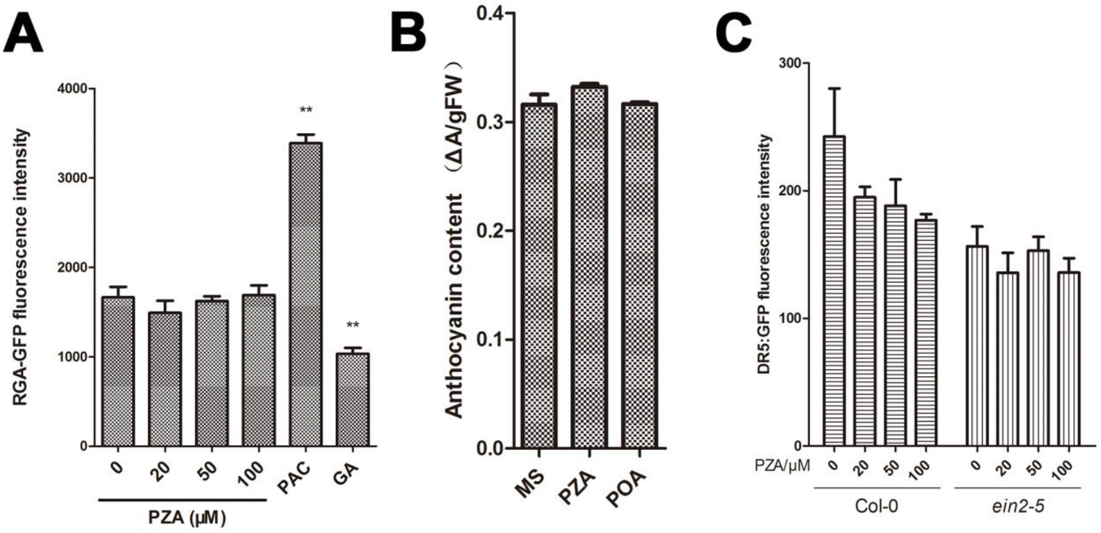

Supplementary Figure 9 PZA or POA does not affect gibberellin response, anthocyanin accumulation or auxin response directly.

(**A**) Quantifications of the GFP fluorescence in the roots of *pRGA::GFP–RGA*. Seedlings were grown on MS treated with different concentrations of PZA (0 μM, 20 μM, 50 μM, and 100 μM), 1 μM PAC and 10 μM GA_3. Bars represent the means (± S.D.) of fifteen seedlings (Student's t test, between PZA-treated and non-PZA-treated seedlings; ** $P < 0.01$).

(**B**) Quantifications of the anthocyanin content of six-day-old Col-0 green seedlings under different treatments. Bars represent the means (± S.D.) of fifteen seedlings.

(**C**) Quantifications of the GFP fluorescence in the roots of *pDR5::GFP/*Col-0 and *pDR5::GFP/ein2-5* under different treatments. Bars represent the means (± S.D.) of fifteen seedlings.

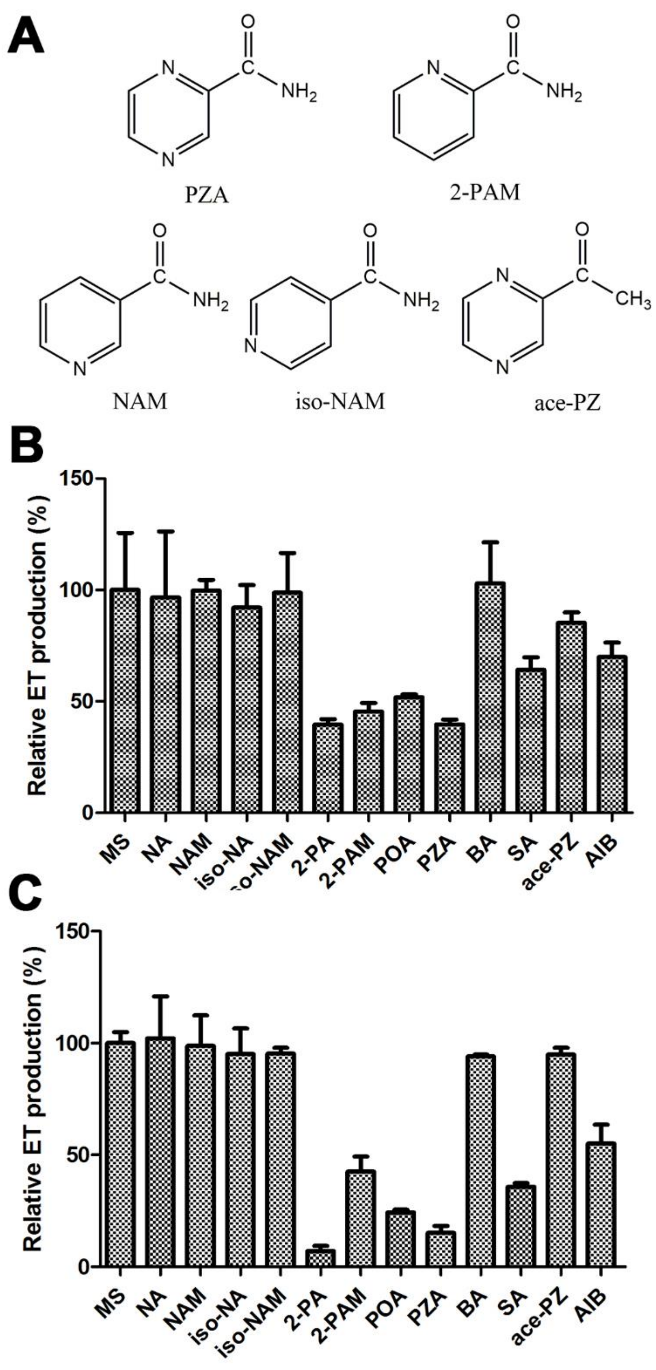

Supplementary Figure 10 Structure-activity relationship of PZA and POA derivatives.

(**A**) Chemical structures of PZA derivatives. 2-PAM, 2-Picolinamide; NAM, Nicotinamide; iso-NAM, iso-Nicotinamide; ace-PZ, Acetylpyrazine
(**B**) & (**C**) Quantification of the relative ethylene production of three-day-old etiolated seedlings of *eto1-2* (**C**) and suspension cultured cells (**D**) with the application of different POA and PZA derivatives (50 μM). Values represent the mean (± S.D.) of three independent treatments.

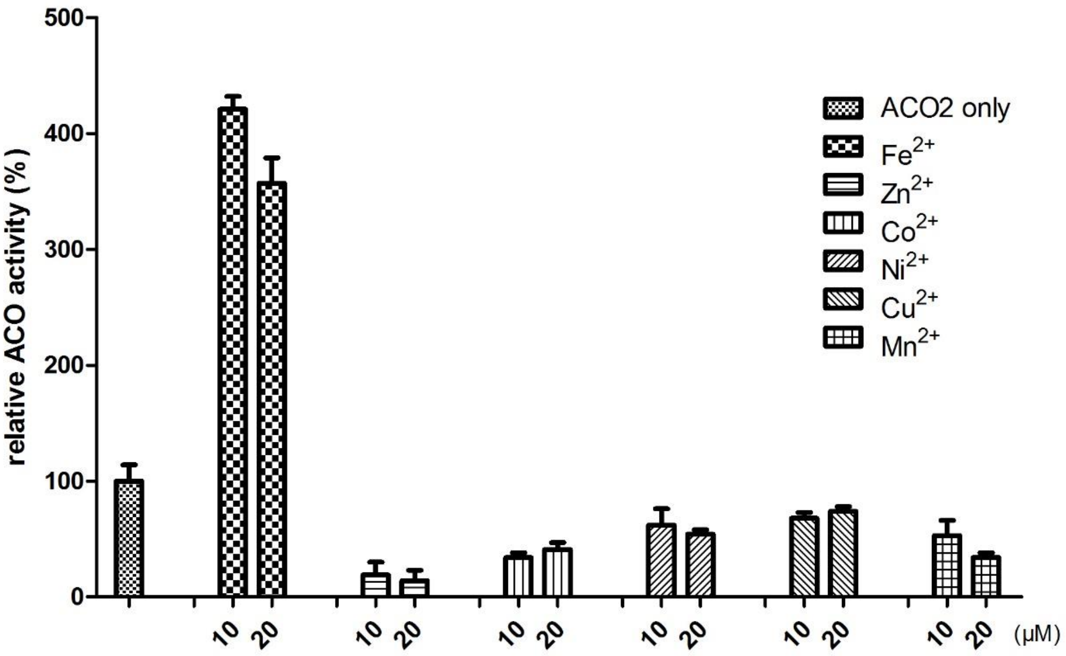

Supplementary Figure 11 The effect of metal ion on the interaction between POA and ACO and the activity of ACO.

Quantifications of the relative AtACO2 activity treated with different divalent metal ions. Values represent the mean (± S.D.) of three replicates, and the experiment was carried out twice with similar results.

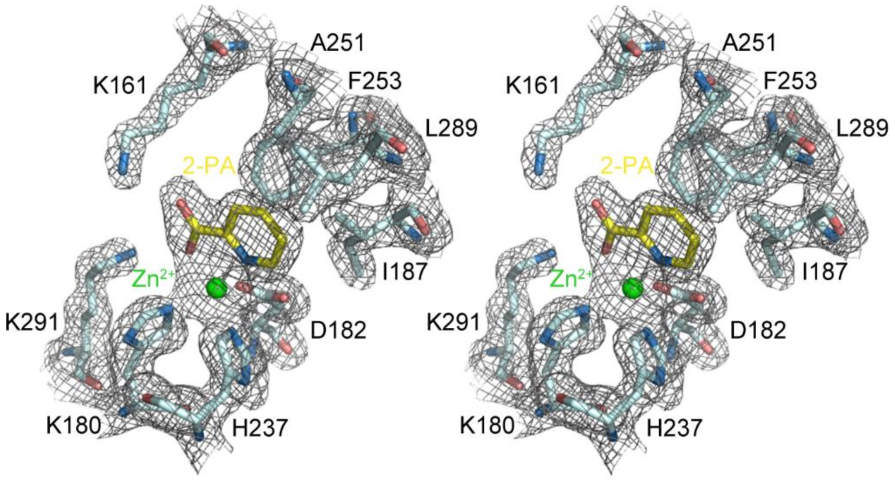

Supplementary Figure 12 A stereo view of the active site of the AtACO2/2-PA complex structure.

Stereo view of 2Fo-Fc electron density map (contoured at 1.5σ) around 2-PA and Zn^{2+} in the active site of AtACO2. 2-PA and relevant residues are shown as yellow and blue sticks, respectively. Zn^{2+} is shown as a green sphere. The electron density map is shown as a gray mesh.

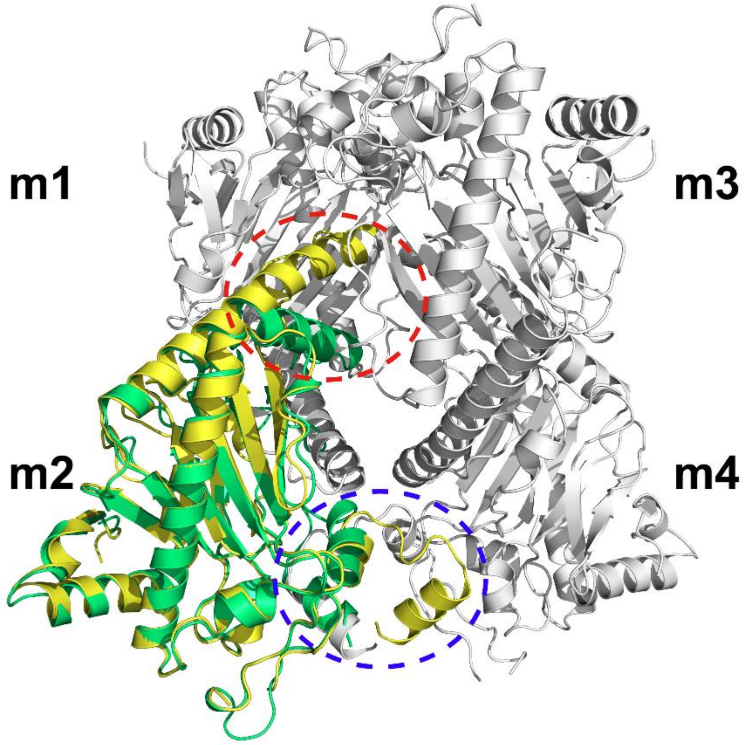

Supplementary Figure 13 Structure comparison of AtACO2 and PhACO.

AtACO2, colored in green, is superimposed onto one molecule of PhACO (yellow) in the PhACO tetramer (labeled as m1-4). The other three molecules of PhACO in the tetramer are shown in white. Different conformations are seen in the regions corresponding to PhACO α3 (red dashed circle) and α11 (blue dashed circle).

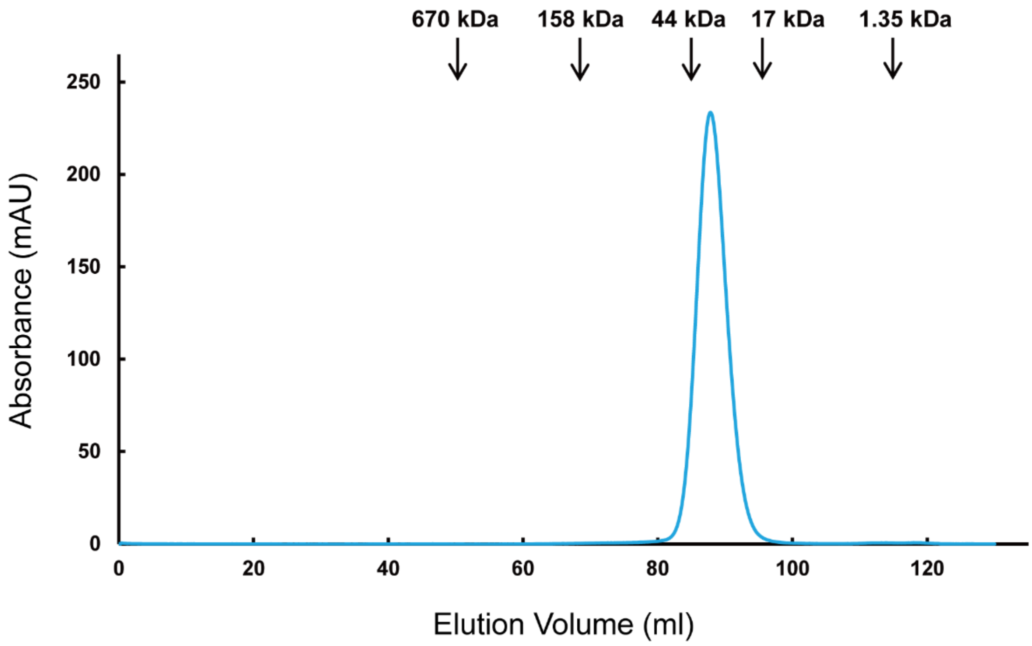

Supplementary Figure 14 Size exclusion chromatography analysis of AtACO2.

Size exclusion chromatography experiment was performed on a Superdex 200 column (GE healthcare). Compared with the molecular weight standard, AtACO2 (~31 kDa) eluted at a position consistent with the size of a monomer.

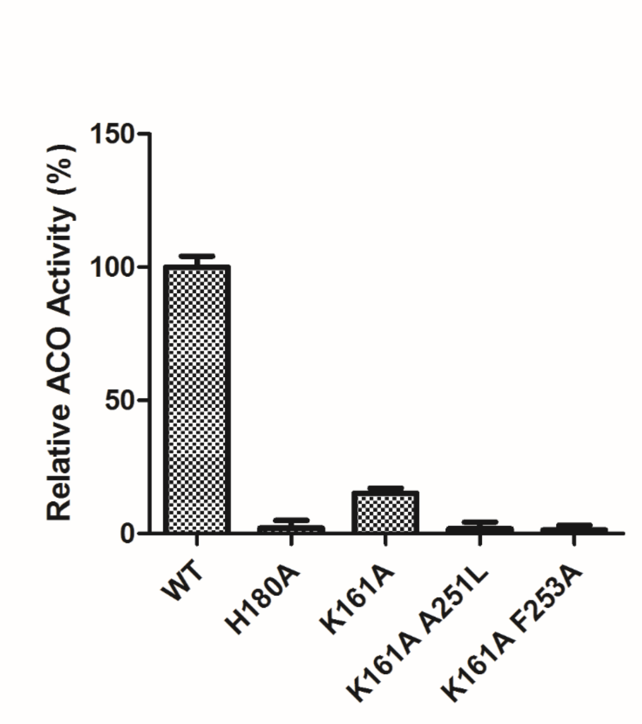

Supplementary Figure 15 POA-binding defective mutants of AtACO2 have reduced or abolished activity.

Quantification of the relative ACO activity (compared to WT) of different ACO mutants. Bars represent the means (± S.D.) of three independent replicates, and the experiment was carried out twice with similar results.

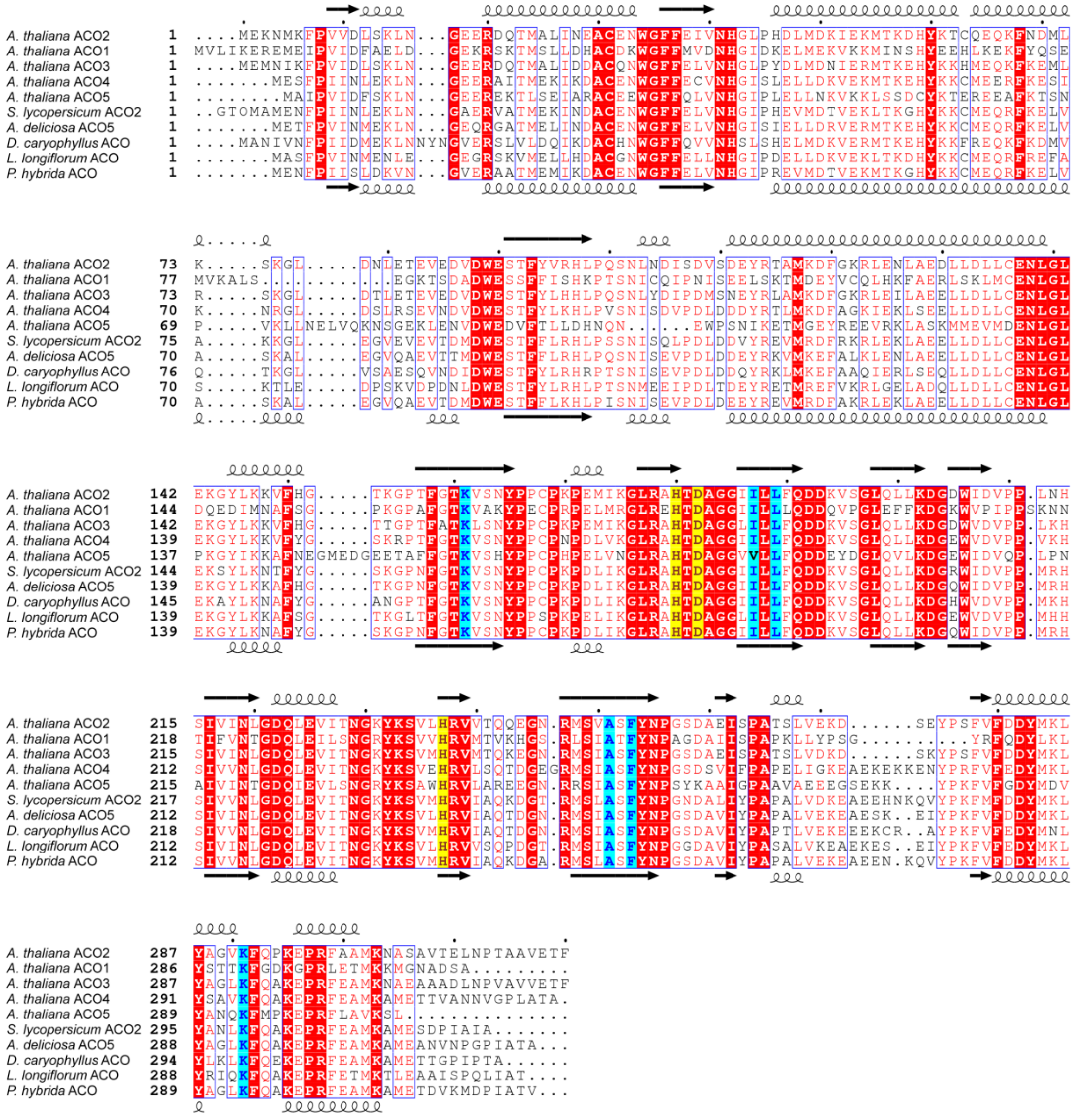

Supplementary Figure 16 Sequence alignments of ACO homologs.

Multiple sequence alignment of selected ACO homologs (*S. lycopersicum*: tomato; *A. deliciosa*: kiwifruit; *D. caryophyllus*: carnation: *L. longiflorum*: easter lily) is performed using clustalW and figure generated using Espript. Secondary structural elements of AtACO2 and PhACO are shown above and below the sequence block, respectively. Highly conserved residues are shown in red boxes. Residues involved in Fe^{2+}/Zn^{2+}-binding are highlighted with yellow color. Other residues essential for the interaction with POA/2-PA are highlighted with blue color.
